# Editorial: Emerging technologies in infectious disease treatment, prevention and control

**DOI:** 10.3389/fcimb.2022.1096998

**Published:** 2022-11-30

**Authors:** Buket Baddal

**Affiliations:** Department of Medical Microbiology and Clinical Microbiology, Faculty of Medicine, Near East University, Nicosia, Cyprus

**Keywords:** emerging infectious diseases, surveillance, infectious disease prevention and control, disease modeling, diagnostics

Surveillance and tracking of emerging infectious diseases play a critical role for the early identification of public health threats. A number of factors can be attributed to the emergence of new infections including human factors such as increased density of human population, international travel, trade and the interaction between humans and wildlife as well as ecological factors such as drastic changes in climate and agricultural practices. With the recent negative impact of the COVID-19 pandemic on global public health and economies, prioritization of research and development of novel technologies to predict, monitor, detect and prevent such infectious disease threats has become fundamental. In addition, the continued emergence and re-emergence of infectious diseases such as monkeypox, West Nile virus and Ebola infections, is further highlighting the urgent need for novel technologies that could strengthen our capacity to prevent, detect and control potential pandemic threats. The overarching goal of our Research Topic was to reconcile pioneering high-quality research manuscripts which have used innovative technologies for infectious diseases pathogen detection, identification and investigation of diagnostic markers.

In the first article contributing to our Research Topic (Bang et al.), the authors have utilized nanopore whole-genome sequencing (WGS) technology to investigate a laboratory-acquired Zika virus (ZIKV) infection during research vaccine development. Laboratory-acquired infections (LAIs) can often be undetected due to the low discriminatory power of recommended laboratory tests used for the detection of the virus, such as real-time reverse transcriptase PCR assays or antibody-based assays. In this study, ZIKV isolated from the urine samples of an infected laboratory personnel was sequenced along with the two strains, KU955591.1 and KX446950.2, which were used in the vaccine development. The authors showed that the MinION (Oxford Nanopore Technologies) DNA sequencing platform which generates long reads, is a promising technology for the identification of the link between viral strains used in vaccine development and the strain present in the infected patient. Together with a literature review of LAIs that occurred from 2011 to 2020, the study concludes that in most cases of LAIs, laboratory exposure is not investigated, and that sequencing technologies could facilitate the investigation of such exposures. This phenomenon was highlighted also by the previous studies which demonstrated the value of WGS as a tool for identifying LAIs (Lim et al., 2004; Alexander et al., 2016; Smith et al., 2017).

The second article focused on the association between gut dysbiosis and sepsis-induced myocardial dysfunction (SIMD) in patients with sepsis or septic shock (Chen et al.). The authors exploited 16S rRNA gene sequencing in order to assess the compositions of the gut microbiota in septic patients with or without myocardial injury, and to evaluate the relative abundance of microbial taxa, clinical indicators and clinical outcomes in the context of SIMD. Importantly, the authors demonstrated that the beta-diversity of the gut microbiota was significantly different between the SIMD patients and non-SIMD subjects, with the microbial abundance of *Klebsiella variicola* being notably higher in the SIMD group. Authors concluded that K. *variicola* could serve as a potential biomarker of SIMD.

Novel platforms and techniques for the accurate laboratory detection of infectious pathogens are invaluable for the rapid clinical diagnosis and effective treatment. Traditional detection methods for the human pathogen *Mycoplasma pneumoniae* include culture, serological testing and polymerase chain reaction (PCR)-based methods which can have low sensitivity and specificity depending on the selected commercial kit, or may require complex equipment and trained personnel. Xiao et al. designed a novel loop-mediated isothermal amplification (LAMP)-based assay combined with a nanoparticle-based lateral flow biosensor (LAMP-LFB) for the rapid and sensitive detection of *Mycoplasma pneumoniae*. The authors developed the LAMP-LFB assay with a set of six oligonucleotide primers targeting different regions of the community-acquired respiratory distress syndrome (CARDS) toxin gene which produces results within an hour. By comparing the assay results with the real-time PCR method, and validating the assay with 100 nasopharyngeal swab samples obtained from children suspected of having *M. pneumoniae* infection, authors have demonstrated that the LAMP-LFB assay was a rapid, highly sensitive and specific test which can be applied at the point-of-care settings and basic medical facilities in rural areas.

Within the same notion of harnessing new technologies to advance infectious disease diagnosis, Sun et al. have developed a real-time multiple cross displacement amplification assay (MCDA) for the rapid and sensitive detection of *Haemophilus influenzae* in clinical specimens. The current laboratory diagnosis of *H. influenzae* mainly employs culture in which results can be influenced by prior antibiotic use, antigen tests which can be prone to inconsistencies as well as PCR tests and loop-mediated isothermal amplification (LAMP) assays which can be time-consuming and labor-intensive. In this innovative study, authors have devised an assay which integrated MCDA strategy with real-time fluorescence detection that avoids subjective errors and contamination of clinical samples, and offers a more sensitive detection rate of 10 CFU per reaction over 500 CFU per reaction in the LAMP test within 40 minutes.

Next-generation sequencing (NGS) technology has rapidly evolved and gained popularity over the last 15 years due to its high sensitivity for pathogen detection. Compared to the traditional culture methods, NGS, an emerging powerful innovative technology, has advanced infection diagnosis. In their contributed work to this Research Topic, Cao et al. present NGS as a valuable platform for pathogen detection in human blood. Interestingly, the authors were able to identify the causative agent in sepsis patients using NGS, which is applicable to the detection of rare pathogens and fastidious bacteria as well as mixed infections. The study concluded that the combination of NGS with albumin level measurements in suspected sepsis patients can improve the sensitivity and specificity of diagnosis, which remains a challenge in modern intensive care medicine. This is consistent with previous studies showing that NGS technology is advantageous for the diagnosis of infecting pathogens in sepsis, particularly for microorganisms that are currently difficult or impossible to culture (Brenner et al., 2018; Ing-Kit Lee et al., 2022). We believe that exponential progress through technology-driven innovation will continue to guide the next era of infectious disease research.

## Author contributions

The author confirms being the sole contributor to this editorial and has approved it for publication.

## Acknowledgements

We acknowledge all authors that have contributed to the success of our Research Topic.

## Conflict of interest

The author declares that the research was conducted in the absence of any commercial or financial relationships that could be construed as a potential conflict of interest.

## Publisher’s note

All claims expressed in this article are solely those of the authors and do not necessarily represent those of their affiliated organizations, or those of the publisher, the editors and the reviewers. Any product that may be evaluated in this article, or claim that may be made by its manufacturer, is not guaranteed or endorsed by the publisher.
